# Are Canadian Clinical Practice Guidelines Accounting for Adults With Multiple Chronic Diseases? A Systematic Review

**DOI:** 10.1111/jep.70143

**Published:** 2025-06-10

**Authors:** Olivia L. Tseng, Shanjot Brar, Martin Dawes, Hetesh Ranchod, Diane Lacaille, Victoria C.H. Su, Craig Mitton

**Affiliations:** ^1^ Department of Family Practice University of British Columbia Vancouver British Columbia Canada; ^2^ Centre for Clinical Epidemiology & Evaluation Vancouver British Columbia Canada; ^3^ Faculty of Medicine University of British Columbia Vancouver British Columbia Canada; ^4^ Vancouver Coastal Health Vancouver British Columbia Canada; ^5^ Division of Geriatric Medicine Providence Health Care Vancouver British Columbia Canada; ^6^ Arthritis Research Canada Vancouver BC Canada; ^7^ Department of Medicine, Faculty of Medicine University of British Columbia Vancouver British Columbia Canada; ^8^ St. Paul's Hospital Providence Health Care Vancouver British Columbia Canada; ^9^ School of Population and Public Health Vancouver British Columbia Canada

**Keywords:** clinical effectiveness, clinical practice guideline, cost effectiveness, intervention effectiveness, multimorbidity, social determinants of health

## Abstract

**Rationale:**

Recommendations that are equipped with essential and adequate information promote adherence and support evidence‐informed decision‐making, which are crucial attributes of patient‐centered care when caring for patients with multiple coexisting health conditions.

**Aims and Objectives:**

To systematically evaluate the content of recommendations of Clinical Practice Guidelines in Canada.

**Method:**

We searched PubMed, MEDLINE, Embase, and professional organization websites to identify 18 Canadian guidelines addressing 14 diseases prevalent in adults with multimorbidity in nonhospital settings. Two reviewers independently appraised the included guidelines using the international AGREE II tool, extracted 2,509 recommendations and assessed each recommendation to determine the presence of primary health outcomes, as well as secondary demographics and the number of involved diseases. We stratified the findings by potential modifiers: level of evidence (LOE) and type of recommendations (e.g., screening and diagnosis).

**Results:**

Half of the guidelines were high‐quality, with all domains scoring 50% or higher. The format and definitions of LOE were found to be heterogeneous. A significant portion focused on a single disease (72%), did not include any demographic information (72), or missed health outcomes (66%). Health outcomes were more frequently addressed in pharmacological (17.6%) and Nonpharmacological (14.5%) management recommendations than in screening (0.7%) and diagnosis (1.1%) recommendations.

**Conclusion:**

There is significant variation in guidelines. For health professionals such as primary care whose patients have multiple conditions, this variation is unacceptable. A centralized guideline development agency would reduce inconsistencies in formatting among guidelines, promoting adherence. Recommendations equipped with adequate information are pivotal in supporting patient‐centered care through evidence‐informed decision‐making.

**PROSPERO registration**: CRD42020105261.

**Abbreviations**
AGREE: Appraisal of Guidelines for Research and Evaluation
C‐CHANGE: Canadian Cardiovascular Harmonized National Guidelines Endeavour
CDSS: Clinical decision‐making support systems
CMA: Canadian Medical Association
EMR: Electronic medical record
LOE: Level of evidence
NICE: National Institute for Health and Care Excellence
NNT: Number needed to treat
PCPs: Primary care physicians
PRISMA: Preferred Reporting Items for Systematic Review and Meta‐analysis Protocols
QALY: Quality‐adjusted life years
SD: Standard deviation
SOR: Strength of recommendation
WHO: World Health Organization

## 1 Introduction

Multimorbidity is an increasingly significant issue that challenges healthcare systems worldwide. One in three individuals is considered multimorbid, meaning they have two or more coexisting long‐term chronic health conditions [1]. The prevalence of multimorbidity is particularly high in North America, where it affects 43% of the population [1]. Despite being high users of healthcare services [2], individuals with multimorbidity often experience poorer health outcomes [3] and a lower quality of life [4]. Their care needs are often complex, encompassing biomedical, psychological, and social aspects. While this patient group continues to grow, their care remains suboptimal. To tackle these pressing challenges, the World Health Organization (WHO) [5] and the National Institute for Health and Care Excellence (NICE) [6] strongly advocate for a patient‐centered approach. This emphasizes the importance of providing only “selected” and “prioritized” care aligning with the personal preferences of multimorbid patients [7]. However, this approach has not been adequately supported by existing clinical practice guidelines due to their known unclear applicability and interpretability.

Clinical practice guidelines are designed to assist clinicians in delivering consistent, high‐quality care to promote patient health [8, 9, 10]. Most guidelines primarily focus on single diseases, largely due to the lack of research evaluating disease‐disease, disease‐drug, and drug‐drug interactions [11]. Applying multiple guideline recommendations simultaneously to manage the various conditions of a multimorbid patient can lead to fragmented care and an increased treatment burden [12, 13, 14]. Furthermore, most guidelines tend to prioritize generalization over specificity, often developing one‐size‐fits‐all guideline recommendations [15]. These broad guideline recommendations frequently overlook key factors that are essential for patient‐centered care, such as demographic variables that influence treatment outcomes, coexisting health conditions that may interact with drugs and diseases, and the balance of benefits and harms critical for shared decision‐making. The absence of these factors reduces the applicability and interpretability of guideline recommendations, leading to poor compliance driven by increased uncertainty and confusion among both providers and patients [15, 16, 17, 18, 19].

The international experts recommended considering applicability and quality of evidence when applying existing evidence to multimorbid patients, including factors such as the demographics of study populations, as well as the benefits and harms [20]. Moreover, several articles have advocated for incorporating factors such as age, race, and sex into guideline development [21, 22, 23]. However, the extent to which guideline recommendations integrate these factors to facilitate patient‐centered care remains unclear, particularly in countries without a centralized guideline organization, such as Canada. This lack of standardization results in noticeable variations in the development process, content, and format of guideline recommendations. Therefore, this study aims to compare the content of Canadian guideline recommendations from a multimorbidity perspective to enhance awareness and identify knowledge gaps.

## 2 Methods

This is a systematic review evaluating Canadian guideline recommendations from a multimorbidity perspective. We registered the study protocol at PROSPERO (registration number CRD42020105261, available at: https://ww.crd.york.ac.uk/PROSPERO/ *)*. We reported study results using criteria from the Preferred Reporting Items for Systematic Review and Meta‐analysis Protocols (PRISMA) [24]. Two independent reviewers (SB/DB, OT) selected studies and extracted data at each stage. Reviewers SB and DB alternated as the first reviewers. The reviewers resolved disagreements by discussion.

A Clinical Practice Guideline is a text document that includes one or more evidence‐based guideline recommendations primarily related to a disease, such as diabetes or hypertension. Each guideline recommendation is a brief statement formulated with research evidence extracted from literature reviews or experts' opinions when research evidence is lacking [25, 26]. Each recommendation answers a clinical question of when and how to screen, prevent, investigate, treat or manage the target disease [8]. The quality of a recommendation is often rated with a level of evidence (LOE), strength of recommendation (SOR) or both [27]. A LOE reflects the methodological quality, validity, and reliability of evidence that forms the recommendation. A SOR indicates the magnitude of outcomes, effects of what would occur when following the recommendation.

### 2.1 Search Strategy

We included the MEDLINE, Embase, and CINAHL databases in our search for their broad healthcare research content and excluded the CENTRAL data set for controlled trials. We consulted a UBC senior librarian with expertise in clinical systematic reviews to build search strategies. The first author (OT) searched three databases with the keywords and MeSH terms extracted from sample articles related to the study (Appendix I). The search result was then limited to the English language and the year of publication, 2010–2018. We validated the search strategies using 10 sample articles and examined 20% of articles excluded and included when adding or removing keywords or MeSH terms. We also hand‐searched the Canadian Medical Association (CMA) guideline database and Canadian professional organization websites. The search was initially completed in April 2018, which was then revised and executed again on August 26, 2020.

### 2.2 Study Selection

**Figure 1:**
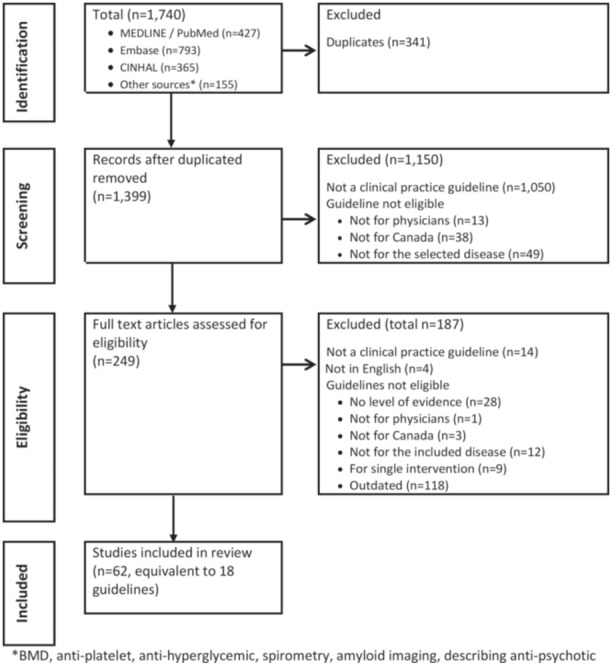
PRISMA flow sheet. *BMD, antiplatelet, anti‐hyperglycemic, spirometry, amyloid imaging, describing anti‐psychotic.

Articles identified by the search strategy were exported to Endnote X9 reference management software (Clarivate Analytics, Philadelphia), where duplicates were removed. The remaining articles were then exported to an Excel worksheet for title/abstract screening, followed by full article reviews (Figure 1).

We included articles that were the most current guidelines developed for Canadian physician use, targeted one of 14 pre‐selected common diseases in nonhospital settings, included recommendations graded for LOE, and were primarily for adults aged 18+ (Appendix II). Only Canadian guidelines were selected to align with our study aim and to avoid intercountry variation in guideline format. Adults aged 18+ were selected as they account for most patients with multimorbidity [28]. We excluded guidelines focused on interventions (e.g. catheter‐directed treatment) or drugs (e.g. anti‐hyperglycemic) and articles derived from primary guidelines without LOE assigned. Our limited resources restricted us to 14 pre‐selected diseases that commonly coexist in multimorbidity patients based on our literature review [28, 29].

We noticed two unique situations. First, guidelines for three diseases were published as sequentially numbered chapters in more than one peer‐reviewed article, such as DM (342 pages, 37 articles) [30], depression (seven articles) [31], and RA (two articles) [32, 33]. Considering they were generated simultaneously and related, we collated them by disease for quality appraisal and data extraction. We also searched for single references for these three guidelines to reduce the reference complexity. Second, guidelines for hypertension [34, 35] and stroke [36, 37, 38] were published in more than one article: related subtopics by different author teams from the same organizations in the same calendar year. We appraised each article individually. The scores were similar across guidelines for the same disease. So, we reported averaged scores for each disease and collated them accordingly for data extraction.

### 2.3 Quality Appraisal

We selected the Appraisal of Guidelines for Research and Evaluation (AGREE)‐II criteria [39] for its well‐known reputation in the guideline field. The AGREE tool consists of 23 items categorized into six domains: scope and purpose (Domain 1 (D1), three items), stakeholder involvement (D2, three items), rigor of development (D3, eight items), clarity of presentation (D4, three items), applicability (D5, four items) and editorial independence (D6, two items). Both reviewers (OT and SB) completed online training modules (https://www.agreetrust.org/agree-ii/) before the appraisal. Each reviewer scored a guideline for each item, ranging from 1 (lowest) to seven (highest). Item scores within each domain were used to calculate domain scores in percentage based on the formula “(obtained score – minimal possible score) divided by (maximum possible score – minimum possible score)” [39]. We then generated a mean domain score, an average of six domain scores, for each guideline to represent its overall quality. A guideline was considered high quality if all domains scored at least 50%, a common cut‐off based on Bargeri et al. study [40]. Reviewer agreement (inter‐rater reliability) was assessed by comparing their domain scores using linear weighted Kappa (http://vassarstats.net/kappa.html *)* and defined as fair (kappa=0.21–0.4), moderate (0.41–0.6) or substantial (0.61–0.8) [41].

### 2.4 Data Extraction

The reviewers extracted data onto standardized Excel worksheets, including bibliographic information (author, year), guideline information (disease, new/updated status of the guideline, number of included recommendations, the grading system for recommendation including LOE and SOR, and outcomes) (Appendix III).

A guideline recommendation is defined as a “systematically developed statement to assist practitioners and patient decisions about appropriate health care for specific circumstances” [42]. The recommendations often included the word “recommend and summarized in boxed text, gray‐colored text, summary tables or special paragraphs.

### 2.5 Outcomes

The *primary outcome* was whether a recommendation included health outcome information, either positive (benefits) or negative (harms) (Table 1). We initially focused on common ones, quality‐adjusted life years (QALY, cost‐effectiveness) and the number needed to treat (NNT, clinical effectiveness), which existed in fewer than 1% of the extracted recommendations. So, we expanded to include any health outcomes in the final analysis, ranging from QALY to NNT, incremental cost‐effectiveness ratio (ICER), disease incidence and hospitalizations [45], disease incidence, hospitalizations, and side effects. Reviewers categorized a recommendation as “Y” (Yes) if it included health outcome information.

**Table 1:** Example outcome(s) of guideline recommendations.

Outcome(s)	Example guide recommendation	Guidelines
Age, sex, and health outcome	We recommend that a cardiovascular (CV) risk assessment be completed every 5 years for “men and women” aged “40 to 75” years using the modified Framingham Risk Score or Cardiovascular Life Expectancy Model to guide therapy to “reduce major CV events” (Strong Recommendation; High Quality Evidence).	Dyslipidemia [43]
Coexisting disease (*n* = 2)	For hypertensive patients with “coronary artery disorder (CAD),” but without coexisting systolic heart failure, the combination of an ACE inhibitor and ARB is not recommended (Grade B)	Hypertension [34]
Sex, Ethnicity	Attainment of a healthy body weight before conception should be pro‐ moted among “Indigenous women” to reduce their risk for gestational diabetes mellitus (GDM) (Grade D, Consensus)	Diabetes [30]
Assuming all ages, both sexes and any ethnicity, health outcome	We recommend physical activity to “reduce the risk of developing heart failure” in “all individuals” (Grade A).	Heart Failure [44]
Health outcome	Prophylactic use of anticonvulsant medications in patients with ischemic stroke is not recommended and there is some evidence to suggest possible harm with “negative effects on neurological recovery” (Evidence Level C)	Stroke – acute stroke management [36]

The *secondary outcomes* were the existence of demographics and the number of coexisting diseases (Table 1). When it had demographic information, each recommendation was categorized as “Y” (Yes) for age, gender, sex, and ethnicity, respectively. Age information in a recommendation can be presented as age group (e.g., 40–60 years old, middle‐aged), age threshold (> 60 years old) or descriptive words of adults, older adults, or all individuals. Sex, defined as biological and physiological characteristics [46], was presented as males, females, men and women in this study. Gender, defined as socially and culturally constructed roles [46], was presented as trans‐men, trans‐women, female‐to‐male, male‐to‐female, transgender males and transgender females. To distinguish between sex and gender in this study, men and women were classified into sex but not gender. Reviewers also counted the number of coexisting diseases included in each recommendation.

### 2.6 Data Analysis

We conducted descriptive analyzes of frequency counts and percentages stratified by grades and types of recommendations. Both grade and type factors may influence the recommendation distributions.

However, recommendation grading was inconsistent among guidelines. Most studies adopted or modified a GRADE system to grade recommendations in the format of (SOR, LOE) (Table 2). While SOR focused on effectiveness (health outcomes), LOE primarily evaluated methodological strengths. We developed an artificial four‐tiered system to reclassify the extracted recommendations based on their LOEs to promote comparability. The Reviewers reassigned each recommendation into A (highest LOE in the original guideline), B (second highest), C (third highest) or D (lowest). For Osteoporosis and Substance Abuse guidelines, we condensed their original six LOEs into four by aligning their definitions with other guidelines (Appendix IV). We chose LOE over SOR due to more consistent definitions and levels among the included guidelines. Also, SOR overlapped with the study outcomes in evaluating effectiveness based on health outcomes.

**Table 2:** Characteristics of the included Clinical Practice Guidelines.

Guideline					Grading Recommendation					
							Level of evidence (LOE)		Strength of recommendation (SOR)	
Disease	Year	Organization	Status	Rec (*N*)	System	Format	Levels High‐Low	Factors	Grading High‐Low	Factors
Anxiety [47]	2014	Anxiety Disorders Association of Canada	New	217	Custom system	(LOE) OR SOR (LOE)^1^	1–4	Strength of evidence for the intervention	1st−3rd line, not rec	Level of evidence, clinical trial efficacy, clinical practice effectiveness and side effects
Asthma [48]	2012	CTS	Update	37	CHEST grading system	(SOR, LOE)	A–C, consensus	Methodological quality^2^	Strong, weak, consensus	Benefits versus risk and burden including cost, associated with adherence to the recommendations, morbidity, mortality, and quality of life.
COPD [49]	2017	CTS	Update	23	CHEST grading system	(SOR, LOE)	A–C, consensus	Methodological strength^2^	1, 2, consensus	Strength of supporting evidence and strength of recommendation based on benefits versus risks, harms, and burdens
Coronary Artery Disease [50]	2014	CCS	New	38	GRADE System 2010	(SOR, LOE)	High–very low quality^3^	Strength of evidence^2^	Strong, conditional	Quality of evidence, difference between desirable and undesirable effects, values and preferences and cost^4^ (For diagnostic testing):
Dementia [51]	2015	CTFPHC	Update	1	GRADE system 2013	(SOR, LOE)	High–very low quality^3^	Likely further research is to change our confidence in the estimate of effect	Strong, weak	Quality of supporting evidence, the degree of uncertainty about the balance between desirable and undesirable effects, the degree of uncertainty or variability in values and preferences, and the degree of uncertainty about whether the intervention represents a wise use of resources
Depression [31]	2016	CANMAT	Update	255	Modified GRADE system	(LOE) OR SOR (LOE)^1^	1–4	Methodological strength^2^	1st−3rd line^1^	Level of evidence and clinical support reflecting expert opinions on feasibility, availability, and clinical effectiveness
Diabetes [30]	2018	Diabetes Canada	Update	656	A standardized tool^2^	(SOR, LOE)	1–4	Paper's objective, methodological rigor, susceptibility to bias and generalizability	A–D (strong–weak)	Totality of evidence (relative strength in methodology and study findings), uncertainty for studies with conflicting outcomes and applicability to Canadian population
Dyslipidemia [43]	2016	CCS	Update	66	GRADE system	(SOR, LOE)	High–very low quality^3^	Methodological strength^2^	Strong, conditional	N/A
Heart Failure [44]	2017	CCS	Update	183	GRADE system 2008/2016	(SOR, LOE)	High–very low quality^3^	Methodological strength^2^	Two levels: Strong and weak	N/A
Hypertension [34, 35]^5^	2018	Hypertension Canada	Update	219	Hypertension Canada Grading system	(Custom)	A–D (strong–consensus) Factor: study methodological quality, effects on a hierarchy of validated clinical outcomes (priority given to cardiovascular morbidity and mortality), and that potential benefits must outweigh potential harms			
Osteoporosis [52]	2014	Osteoporosis Canada	Update	14	CTFPHC	(LOE, SOR)	I, II‐1, II‐2, II‐3, III	Methodological strength^2^	A–D, E^6^, L^7^	Strength of supporting evidence^2^
Rheumatoid Arthritis [32, 33]^8^	2012	CRA	New	56	SIGN system	(LOE, SOR)	I–IV	Methodological strength^2^	A–D (strong‐consensus)	Based on strength of supporting evidence and applicability^2^
Stroke [36, 37, 38]^9^	2017‐2018	HSF	Update	283	GRADE system, 2008	(LOE)	A–C, clinical consideration	Methodological strength, benefits versus harms^2^	None	None
Substance use [53]	2018	Canadian Research Initiative in Substance Misuse	New	11	CTFPHC	(LOE, SOR)	I, II‐1, II‐2, II‐3. III	Methodological strength^2^	A–D, E^6^, L^7^	Strength of supporting evidence^2^

Abbreviations: CANMAT, Canadian Network for Mood and Anxiety Treatments; CCS, Canadian Cardiovascular Society; COPD, Chronic obstructive pulmonary disorder; CRA, Canadian Rheumatology Association; CTFPHC, Canadian Task Force on Preventive Health Care; CTS, Canadian Thoracic Society; HSF, Heart and Stroke Foundation; LOE, Level of evidence; Rec, Recommendation; SOR Strength of recommendation; SIGN, Scottish Intercollegiate Guideline Network.

^1^ For drug treatment only.

^2^ No further details.

^3^ Included four levels of evidence: high, moderate, low, and very low.

^4^ Different factors were considered for diagnostic testing, which included bias, consistency and precision of study results, and readily available methods in community practices.

^5^ Included two guidelines in two related subtopics (hypertension and hypertension in pregnancy) by different author teams from the same organization in the same calendar year.

^6^ Against the clinical prevention action.

^7^ Insufficient evidence to make a recommendation; however, other factors may influence decision‐making.

^8^ Included two guidelines that were published in two sequential numbered publications by the same author team.

^9^ Included three guidelines in three related subtopics (acute management, secondary prevention, and telestroke) by different author teams from the same organization in the same calendar year.

The reviewers categorized recommendations into one of the following types.

- Screening (disease screening and prevention and risk factor identification).
- Diagnosis (such as disease criteria, evaluation investigation and prognosis).
- Pharmacological management (prescription drugs).
- Nonpharmacological management treatment (treatments other than medications, such as surgical interventions, referrals, and monitoring with therapeutic goals) or Lifestyle (e.g., exercise, diet, or supplements).

## 3 Results

We identified 1740 unique articles after 341 duplicates were removed (Figure 1). Reviewers excluded 1150 and 187 articles at the title/abstract and full‐text review stages, respectively. A total of 62 articles fulfilled our selection criteria. We collated them into 18 guidelines for quality appraisal and data extraction.

### 3.1 Study Characteristics

The included guidelines were published between 2012 and 2018 and developed by Canadian professional organizations (Table 2). Four of them were new guidelines, while the others were updated ones. The number of recommendations varied significantly, ranging from one (Dementia guideline) to 249 (Diabetes guideline). LOE was graded in heterogeneous formats, such as A–D, 1–4 or high‐moderate‐low‐very low.

### 3.2 Quality Appraisal

Half of the included guidelines were high‐quality, with all domains scoring at least 50%. The mean domain scores ranged from 55 to 96 (Table 3). Most guidelines scored well in the domains of scope and purpose (domain score, 93), clarity of presentation (91), and editorial independence (96). The score of the applicability domain varied significantly across the included guidelines, with the lowest for the osteoporosis guideline [8] and the highest for the asthma guideline (100). Inter‐rater reliability among reviewers (OT, SB) was moderate based on a 0.72 weighted Kappa coefficient (95% CI, 0.68–0.76).

**Table 3:** Quality evaluation using AGREE II.

	Domain Score (%)							
	1: Scope and purpose	2. Stakeholder involvement	3. Rigor of development	4. Clarity of presentation	5. Applicability	6. Editorial Independence	Mean domain score (SD)	Overall
Anxiety, 2014 [47]	94	61	68	81	21	92	69 (27)	L
Asthma, 2012 [48]	100	67	100	100	100	100	94 (14)	H
Chronic obstructive pulmonary disease, 2017 [49]	97	97	79	100	56	100	88 (18)	H
Coronary artery disease, 2014 [50]	81	53	29	97	52	88	67 (26)	L
Dementia, 2012 [51]	97	58	56	94	29	100	73 (29)	L
Depression, 2016 [31]	97	64	60	86	46	100	76 (22)	L
Diabetes, 2018 [30]	94	100	98	100	81	100	96 (7)	H
Dyslipidemia, 2016 [43]	89	67	41	100	35	100	72 (29)	L
Heart failure, 2017 [44]	89	67	64	100	38	100	76 (25)	L
Hypertension, 2018 [34, 35]	97	56	98	97	86	100	89 (17)	H
Osteoporosis, 2010 [52]	92	50	48	60	8	71	55 (28)	L
RA, 2011 [32, 33]	100	100	76	92	76	100	91 (12)	H
Stroke, 2017 [36, 37, 38]	84	94	85	99	80	100	90 (9)	H
Substance use, 2018 [53]	92	72	66	60	65	88	74 (13)	H
Mean (SD)	93 (6)	72 (18)	69 (22)	91 (14)	55 (28)	96 (9)		

Abbreviations: L, low; H, high; RA, Rheumatoid arthritis.

### 3.3 CPG Recommendations

**Figure 2:**
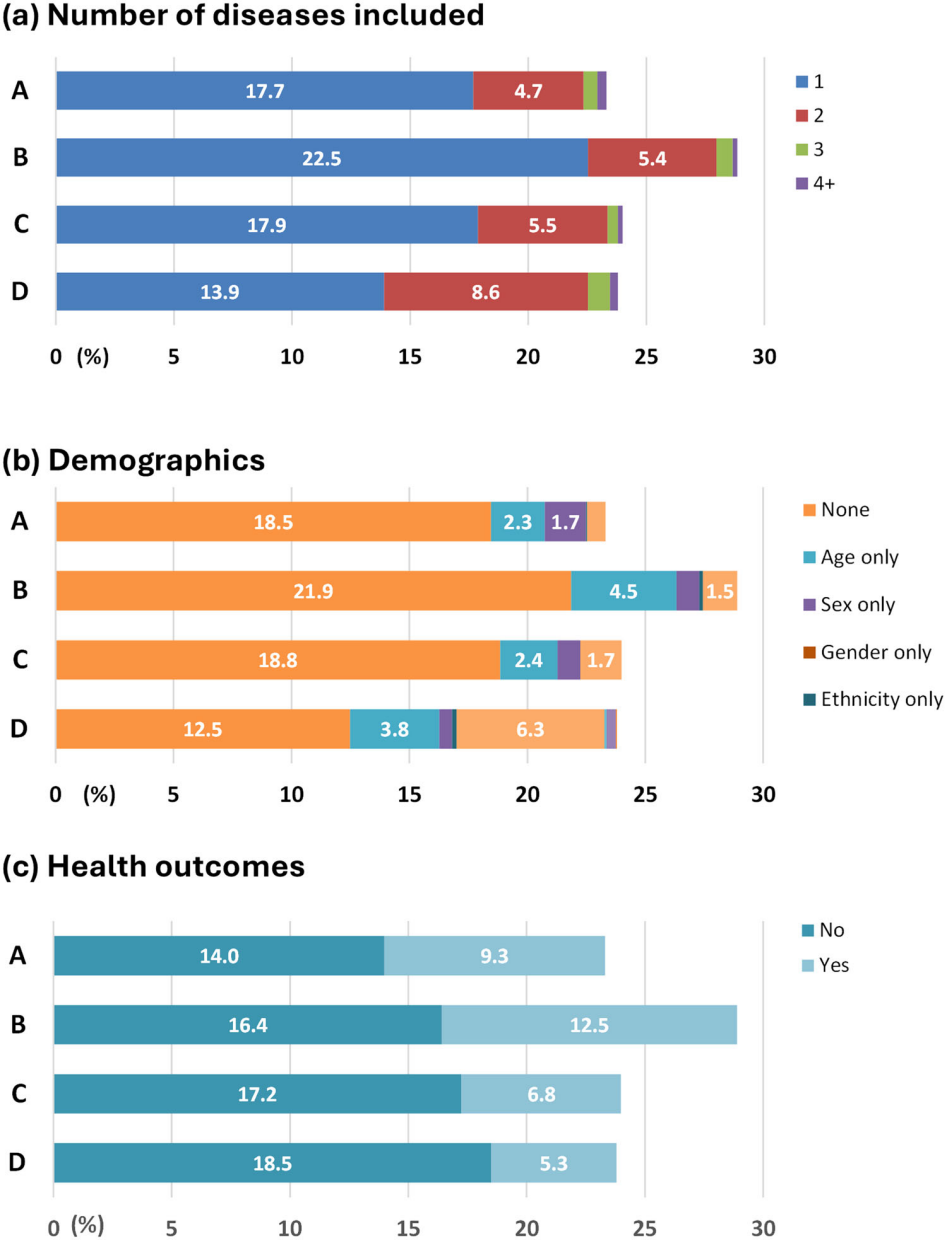
Distribution of guideline recommendations by their grades and study outcomes. (a) Number of dieases included. (b) Demographics. (c) Health outcomes.

**Figure 3:**
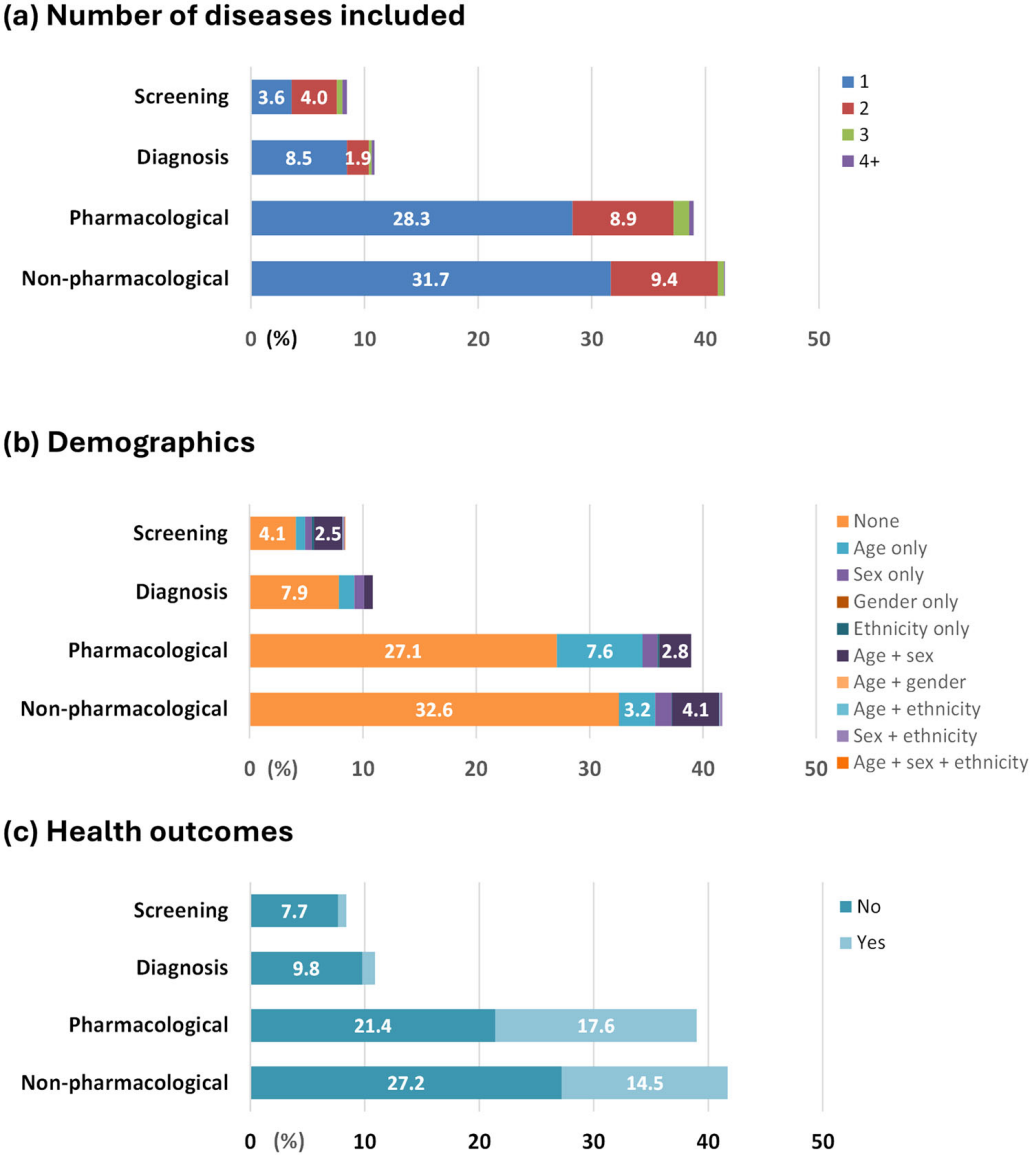
Distribution of guideline recommendations by their types and study outcomes. (a) Number of dieases included. (b) Demographics. (c) Health outcomes.

We extracted and examined a total of 2509 guideline recommendations (Figures 2 and 3, Appendices V and VI). Most of the recommendations mentioned one disease (the one targeted by the guideline, 72%), did not include any demographic information (72%), or did not state health outcomes (66%). When demographic information existed, age‐only or an age‐sex combination was more common than other demographics (i.e., sex only, gender only, ethnicity only, or an age‐ethnicity combination).

When stratified by four levels of evidence (LOE), the distribution of recommendations was roughly equal, ranging from 23% (level A) to 29% (level B). The combination of age and sex was the most common demographic identified in 129 level D recommendations, accounting for 6.3% of all recommendations. Health outcome information was more prevalent in level B recommendations (12.5% of all included recommendations) than in other levels (ranging from 5.3% to 9.3%).

When stratified by type of recommendation, the distribution was skewed. More than 40% of the recommendations were for nonpharmacological management (41%), followed by pharmacological treatments (39%), diagnosis (11%), and screening (9%). Age alone was the most common demographic identified in 66 nonpharmacological management recommendations, accounting for 3.2% of all recommendations. Health outcomes were more frequently stated in pharmacological (17.6%) and nonpharmacological (14.5%) management.

## 4 Discussion

This study comprehensively assessed the content of guidelines for 14 health conditions prevalent among adult patients with multimorbidity. The findings elucidate the challenges posed by the content in delivering patient‐centered quality care. Specifically, over half of the extracted recommendation statements lacked demographic and/or health outcome data. Demographics are necessary for assessing applicability, ensuring recommendations are suitable for individual patients, while health outcomes are essential for patients to make informed decisions aligned with their personal circumstances and preferences. An unforeseen revelation from our study is the disparity in the length, grading system defining Levels of Evidence (LOE), and organization of recommendations within the guidelines. This variability unintentionally adds complexity and confusion to readers navigating through the guidelines.

Our study, employing a similar multimorbidity lens, diverged from the research conducted by Fortin et al. in 2011 [54], particularly in the examination of Canadian guidelines. A notable point of departure lies in the scale of our analysis: guideline versus guideline recommendation. Fortin et al. assessed 16 guidelines using a validated 14‐item checklist, whereas our study evaluated 2500 individual guideline recommendations extracted from 18 guidelines. While the terms guideline and guideline recommendation are often used interchangeably, it is important to distinguish between them. A guideline typically contains multiple guideline recommendations, short statements, or pieces of advice. In our study, for example, the Diabetes guideline included over 600 recommendations, primarily targeted at adults. Of these, 32 recommendations specified age groups, such as ≥ 40, 14–29, 19–64, ≥ 65, < 55, or < 75; more than 200 mentioned adults, older adults, or older adults; and the remaining recommendations lacked any age‐related information. This suggests that the underlying research evidence for these recommendations may be derived from populations that vary by age, gender, sex, ethnicity, and comorbidities. Any recommendations that lack age‐specific information pose a greater challenge for frontline physicians, as it becomes harder to determine their applicability in practice.

Our study findings highlighted barriers identified by prior studies assessing users' experiences, such as lack of applicability, outcome expectancy, and user‐friendliness [15, 17, 18, 55]. Canadian Thoracic Society, a major guideline developer in Canada, advocates “optimal use of language and format to convey recommendations” to promote guide implementation [56]. In this study, the recommendation statements were presented in various formats: integrated in text, as paragraphs in gray backgrounds, within text boxes, or in summary tables. Some statements stood alone, while others were accompanied by clinical questions or relevant research evidence.

Inconsistent recommendation grading further exacerbated confusion. The 18 included guidelines were graded by at least seven systems and presented in four different formats (LOE alone, (SOR, LOE), (LOE, SOR), or SOR (LOE)). Upon closer examination, we noticed the definitions of LOE and SOR also varied from one guideline to another. What may be classified as level‐A evidence in one guideline could be downgraded to level‐B in another. This inconsistency undermines providers' confidence in applying guideline recommendations, which is a barrier to implementing guidelines.

Individualized care is critical when caring for patients with multimorbidity, especially old adults with limited life expectancy. The process involves balancing positive and negative health outcomes (benefit and harm), prioritizing coexisting diseases, and aligning care options with personal preferences [20]. Although our study identified health outcome information in 33% of the extracted recommendations, they often lack quantitative effects to support decision‐making. An example was ‘to reduce hospital admission,’ which did not specify a rate to quantify the reduction. The omission of the rate was intentional, as guidelines often extrapolate evidence from trial participants to larger populations. However, this generalization process hinders patient‐centered care.

In addition to content and format issues that our study demonstrated, there have been efforts to promote better utilization of Clinical Practice Guidelines for multimorbid patients. One approach entails the harmonization of recommendations across individual or regional guidelines to mitigate inconsistencies. An example is the Canadian Cardiovascular Harmonized National Guidelines Endeavour (C‐CHANGE) [57]. Its 2022 version organized 83 recommendations related to multimorbidity based on disease combinations, such as the triad of obesity, diabetes, and hypertension [57]. Unfortunately, it encountered challenges related to inconsistent formatting and variation in evidence grading, thereby warranting further refinement and standardization. Concurrently, another significant effort centers on leveraging computer technology, including machine learning. Guideline knowledge is being integrated into clinical decision support systems (CDSSs), integrated with electronic health record data or incorporated into a Health Information System [58]. Despite their potential for positively impacting patient care, CDSSs encounter limitations concerning data integration and operational efficiency [59]. More recently, there is growing interest in using artificial intelligence to search databases, extract relevant information and summarize advice that is personalized [60]. However, artificial intelligence remains in its infancy, with potential biases and misleading results. Further studies are needed to resolve these limitations.

## 5 Limitations

The study had a few limitations. Firstly, we only focused on Canadian guidelines to highlight the need for a centralized guideline development agency. Our study findings could potentially be applicable to other countries with similar settings and multiple independent guideline developers. Secondly, this study was limited to 14 diseases due to resource constraints. Certain common health conditions, such as obesity, were not included. However, our study successfully demonstrated the inconsistent format among 18 guidelines. Adding more guidelines to this study will not meaningfully alter our findings and conclusions. Lastly, the guidelines we selected were developed between 2010 and 2018, which may be outdated and may not fully reflect content issues addressed in more recent guidelines. However, we expect format variations to persist as long as Canada lacks a centralized organization to standardize both the format and content.

## 6 Conclusion and Implication

Our findings underscore two key issues. Firstly, the absence of a centralized guideline development agency can result in an inconsistent process for distilling research evidence into recommendations, leading to variations in recommendation formats. This lack of uniformity has the potential to confuse both patients and providers, discouraging adherence to recommendations. Secondly, a significant portion of the Clinical Practice Guidelines lacked sufficient information to support evidence‐informed decision‐making, which is fundamental to patient‐centered care.

## Conflicts of Interest

The authors declare no conflicts of interest.

## Originality of Work and Previous Presentation of Manuscript

This study is completely original. A poster was virtually presented in North America Primary Care Research Group Conference held in San Francisco, California, USA on November 20–24, 2020.

## Supporting information

Supplemental info.

## Data Availability

The data that support the findings of this study are available on request from the corresponding author. The data are not publicly available due to privacy or ethical restrictions.
