# Supplementary material for: Are Canadian Clinical Practice Guidelines Accounting for Adults With Multiple Chronic Diseases? A Systematic Review
Source: J Eval Clin Pract. 2025 Jun 10;31(4):e70143. doi: 10.1111/jep.70143 (PMC12150902; doi:10.1111/jep.70143)
Supplement: Supplementary file 1 — Supplemental info. [file JEP-31-0-s001.docx]

## Appendix I: Ovid Medline search strategy

| 1. Clinical practice guideline/ | 27753 |
| --- | --- |
| 2. Evidence-based practice/ | 10296 |
| 3. Professional Practice/st [Standards] | 2520 |
| 4. Practice Guidelines as Topic/ | 120131 |
| 5. Practice Patterns, Physicians'/st [Standards] | 6576 |
| 6. (clinical adj3 (guideline or protocol)).ti,ab,kf. | 12462 |
| 7. 1 or 2 or 3 or 4 or 5 or 6 | 171252 |
| 8. Canada/ | 93100 |
| 9. Canad?.mp. | 148605 |
| 10. Canadian.ti. | 22767 |
| 11. 8 or 9 or 10 | 154284 |
| 12. Hypertension/ | 235753 |
| 13. Dyslipidemias/ | 11804 |
| 14. Coronary Artery Disease/ | 63083 |
| 15. Heart failure/ | 119925 |
| 16. Cardiovascular Diseases/ | 150360 |
| 17. Diabetes Mellitus/ | 118376 |
| 18. Stroke/ | 103591 |
| 19. Intracranial Arteriosclerosis/ | 6108 |
| 20. Dementia/ | 51672 |
| 21. Alzheimer Disease/ | 95075 |
| 22. Cerebrovascular Disorders/ | 46541 |
| 23. Osteoporosis/ | 44538 |
| 24. Arthritis, Rheumatoid/ | 99799 |
| 25. Asthma/ | 126202 |
| 26. Pulmonary Disease, Chronic Obstructive/ | 40298 |
| 27. Depression/ | 120927 |
| 28. Depressive Disorder/ | 73186 |
| 29. Substance-Related Disorders/ | 96060 |
| 30. Mental Health/ | 39541 |
| 31. Hypertension.ti,ab,kf. | 395661 |
| 32. Dyslipidemia.ti,ab,kf. | 28506 |
| 33. Stroke.ti,ab,kf. | 248384 |
| 34. Cerebral Infarction/ | 22039 |
| 35. Dementia.ti,ab,kf. | 112487 |
| 36. Anxiety.ti,ab,kf. | 197153 |
| 37. Depression.ti,ab,kf. | 345025 |
| 38. Depressive.ti,ab,kf. | 119578 |
| 39. Asthma.ti,ab,kf. | 150709 |
| 40. COPD.ti,ab,kf. | 47354 |
| 41. "Chronic obstructive pulmonary".ti,ab,kf. | 50532 |
| 42. "Substance use".ti,ab,kf. | 37209 |
| 43. 11 or 12 or 13 or 14 or 15 or 16 or 17 or 18 or 19 or 20 or 21 or 22 or 23 or 24 or 25 or 26 or 27 or 28 or 29 or 30 or 31 or 32 or 33 or 34 or 35 or 36 or 37 or 38 or 39 or 40 or 41 | 2276076 |
| 44. 7 and 10 and 42 | 913 |
| 45. Limit 43 to English language | 895 |
| 46. Conference paper/ or conference abstract/ or "conference review"/ or symposium/ or workshop/ or editorial/ or letter/ or note/ | 1668661 |
| 47. 44 not 45 | 848 |
| 48. Limit 46 to yr="2010 - 2018" | 427 |

## Appendix II: Inclusion and Exclusion Criteria based on the Population-Concept-Context framework

|  | **Inclusion Criteria** | **Exclusion Criteria** |
| --- | --- | --- |
| Population | - Adults aged 18 and over | Participants under 18 years of age |
| Concept | Guidelines   - For Canadian physician use - Targeting one of the 14 pre-selected diseases - In non-hospital settings - Including guideline recommendations graded for level of evidence | Guidelines   - Not for Canadian physicians - Not targeting one of the 14 pre-selected diseases - Not targeting diseases but interventions or drugs - In hospital or facility settings - Derived from primary guidelines without level of evidence assigned |
| Context | - Publications time is limited to 2010-2018 - Articles are limited to English-language | - Publication time prior to 2010 - Non-English guidelines |

## Appendix III: Data Extraction Sheet Template and example data

| **Data Field** | **Example** |
| --- | --- |
| Reviewer |  |
| Guideline (Disease, Year) | *Hypertension, 2018* |
| Chapter / Sections | 1. *Accurate measurement* |
| No, page | *509* |
| No, recommendation | *1* |
| Recommendation | *Health care professionals who have been specifically trained to measure BP accurately should assess BP in all adult patients at all appropriate visits to determine cardiovascular risk and monitor antihypertensive treatment* |
| Type, advice | *Diagnosis* |
| Grade | *D* |
| Strength of recommendation | *NA* |
|  |  |
| Recommendation content |  |
| Age | *Adult* |
| Gender | *NA* |
| Sex | *NA* |
| Ethnicity | *NA* |
| Clinical effectiveness, NNT | *NA* |
| Clinical effectiveness, any | *NA* |
| Cost effectiveness, years of quality | *NA* |
| Cost effectiveness, any | *NA* |
| Harms | *NA* |
| No of the listed disease | *NA* |

## Appendix IV: summary / comparison of level of evidence

| **Guideline** | **Grading system** | **Level of Evidence** | | | |
| --- | --- | --- | --- | --- | --- |
|  |  | **A** | **B** | **C** | **D** |
| Anxiety  (54) | Custom system | Meta-analysis or at least 2 randomized controlled trials (RCTs) that included a placebo condition; treatment studies in which randomized comparisons are available | At least 1 RCT with placebo or active comparison condition; treatment studies in which randomized comparisons are available | Uncontrolled trial with at least 10 subjects; involving epidemiological or risk factors primarily arise from observational studies | Anecdotal reports or expert opinion; consensus opinion based on evidence from various data source |
| Asthma  (55) | CHEST Grading system | RCTs without important limitations or  overwhelming evidence from observational  studies | RCTs with important limitations (inconsistent  results, methodological flaws, indirect or  imprecise) or exceptionally strong evidence  from observational studies | Observational studies or case series | Consensus |
| COPD  (56) | GRADE System | Consistent evidence from randomized controlled trials without important limitations or exceptionally strong evidence from observational studies. | Evidence from randomized controlled trials with important limitations (inconsistent results, methodologic flaws, indirect or imprecise), or very strong evidence from observational studies. | Evidence for at least one critical outcome from observational studies, case series, or from randomized, controlled trials with serious flaws or indirect evidence. | Consensus-based |
| Coronary Artery Disease  (57) | GRADE System | Future research unlikely to change confidence in estimate of effect, e.g., multiple well-designed, well-conducted clinical trials | Further research likely to have an important impact on confidence in estimate of effect and may change the estimate, e.g., limited clinical trials, inconsistency of results or study limitations | Further research very likely to have a significant impact on the estimate of effect and is likely to change the estimate, e.g., small number of clinical studies or cohort observations | The estimate of effect is very uncertain; e.g./, case studies, consensus opinion |
| Dementia 2015  (58) | GRADE System | NA | NA | NA | NA |
| Depression 2016  (31) | Modified GRADE System | Meta-analysis with narrow confidence intervals and/or 2 or more RCTs with adequate sample size, preferably placebo controlled | Meta-analysis with wide confidence intervals and/or 1 or more RCTs with adequate sample size, plus clinical support | Small-sample RCTs or nonrandomized, controlled prospective studies or case series or high-quality retrospective studies or higher, plus clinical support | Expert opinion, or consensus |
| Diabetes  (30) | Custom system, diagnosis | a) independent interpretation of test results (without  knowledge of the result of the diagnostic or gold  standard); b) Independent interpretation of the diagnostic  standard (without knowledge of the test result); c) Selection of people suspected (but not known) to have the disorder; d) Reproducible description of both the test and diagnostic standard; e) At least 50 patients with and 50 patients without the disorder | Meets 4 of the Level 1 criteria | Meets 3 of the Level 1 criteria | Meets 1 or 2 of the Level 1 criteria |
|  | Custom system, treatment and prevention | Level 1A  Systematic overview or meta-analysis of high-quality  RCTs  a) Comprehensive search for evidence; b) Authors avoided bias in selecting articles for inclusion; c) Authors assessed each article for validity; d) Reports clear conclusions that are supported by the data and appropriate analyses  OR  Appropriately designed RCT with adequate power to  answer the question posed by the investigators a) Patients were randomly allocated to treatment groups; b) Follow up at least 80% complete; c) Patients and investigators were blinded to the treatment*; d) Patients were analyzed in the treatment groups to which they were assigned; e) The sample size was large enough to detect the  outcome of interest  Level 1B Non-randomized clinical trial or cohort study with indisputable results | RCT or systematic overview that does not meet Level 1  criteria | Non-randomized clinical trial or cohort study;  systematic overview or meta-analysis of level 3  studies | Other |
|  | Custom system, prognosis | a) Inception cohort of patients with the condition of  interest, but free of the outcome of interest; b) Reproducible inclusion/exclusion criteria; c) Follow up of at least 80% of subjects; d) Statistical adjustment for extraneous prognostic factors (confounders); e) Reproducible description of outcome measures | Meets criterion a) above, plus 3 of the other 4 criteria | Meets criterion a) above, plus 2 of the other criteria | Meets criterion a) above, plus 1 of the other criteria |
| Dyslipidemia 2016  (52) | GRADE System | NA | NA | NA | NA |
| Heart Failure  (53) | GRADE System  2008/ 2016 | Further research is very unlikely to change our confidence in the estimate of effect | Further research is likely to have an important impact on our confidence in the estimate of effect and may change the estimate | Further research is very likely to have an important impact on our confidence in the estimate of effect and is likely to change the estimate | Any estimate of effect is very uncertain |
| Hypertension  (34) | Hypertension Canada Grading system | Recommendations are based on randomized trials (or systematic reviews of trials) with high levels of internal validity and statistical precision, and for which the study results can be directly applied to patients because of similar clinical characteristics and the clinical relevance of the study outcomes | Recommendations are based on randomized trials, systematic reviews or pre- specified subgroup analyses of randomized trials that have lower precision, or there is a need to extrapolate from studies because of differing populations or reporting of validated intermediate/surrogate outcomes rather than clinically important outcomes | Recommendations are based on trials that have lower levels of internal validity and/or precision, or trials reporting unvalidated surrogate outcomes, or results from non-randomized observational studies | Recommendations are based on expert opinion alone |
| Osteoporosis  (59) | Canadian Task Force on Preventative Health Care | I: Evidence obtained from at least one properly randomized controlled trial | II-1: Evidence from well-designed controlled trials without randomization | II-2: Evidence from well-designed cohort (prospective or retrospective) or case–control studies, preferably from more than one centre or research group  II-3: Evidence obtained from comparisons between times or places with or without the intervention. Dramatic results in uncontrolled experiments (such as the results of treatment with penicillin in the 1940s) could also be included in this category | III: Opinions of respected authorities, based on clinical experience, descriptive studies, or reports of expert committees |
| Rheumatoid Arthritis  (32) | Scottish Intercollegiate Guideline Network (SIGN) System | Meta-analyses, systematic reviews of RCT, or individual RCT | Meta-analysis, systematic reviews of observational studies (cohort/case B control studies), or individual observational studies OR RCT subgroup/post-hoc analyses | Nonanalytic studies, e.g., case reports, case series | Expert opinion |
| Stroke  (37) | GRADE System 2008 | Evidence from a meta-analysis of randomized controlled trials or consistent findings from two or more randomized controlled trials. Desirable effects clearly outweigh undesirable effects or undesirable effects clearly outweigh desirable effects. | Evidence from a single randomized controlled trial or consistent findings from two or more well- designed nonrandomized and/or non-controlled trials, and large observational studies. Desirable  effects outweigh or are closely balanced with undesirable effects or undesirable effects outweigh or are closely balanced with desirable effects. | Writing group consensus and/or supported by limited research evidence. Desirable effects outweigh or are closely balanced with undesirable effects or undesirable effects outweigh or are closely balanced with desirable effects, as determined by writing group consensus. Recommendations assigned Level-C evidence may be key system drivers supporting other | Reasonable practical advice provided by consensus of the writing group on specific clinical issues that are common and/or controversial and lack research evidence to guide practice. |
| Substance Use  (60) | Canadian Task Force on Preventive Health care: level of evidence, strength of recommendation | I: Evidence obtained from at least one properly randomized controlled trial | II-1: Evidence from well-designed controlled trials without  randomization | II-2: Evidence from well–designed cohort (prospective or retrospective) or case–control studies, preferably from more than one centre or research group  II-3: Evidence obtained from comparisons between times or places with or without the intervention. Dramatic results in uncontrolled experiments (such as the results of treatment with  penicillin in the 1940s) could also be included in this category | III: Opinions of respected authorities, based on clinical experience,  descriptive studies, or reports of expert committees |

## Appendix V: Distribution of recommendations by their grades and study outcomes

|  |  | | **Level of evidence** | | | | | | | |
| --- | --- | --- | --- | --- | --- | --- | --- | --- | --- | --- |
|  | **Total** | | **A** | | **B** | | **C** | | **D** | |
|  | N | (%) | N | (%) | N | (%) | N | (%) | N | (%) |
| **Total** | 2059 | (100) | 480 | (23) | 595 | (29) | 494 | (24) | 490 | (24) |
| **Diseases included** |  |  |  |  |  |  |  |  |  |  |
| 1 | 1483 | (72.0) | 364 | (17.7) | 465 | (22.6) | 368 | (17.9) | 286 | (13.9) |
| 2 | 499 | (24.2) | 96 | (4.7) | 112 | (5.4) | 113 | (5.5) | 178 | (8.6) |
| 3 | 54 | (2.6) | 12 | (0.6) | 14 | (0.7) | 9 | (0.4) | 19 | (0.9) |
| 4+ | 23 | (1.1) | 8 | (0.4) | 4 | (0.2) | 4 | (0.2) | 7 | (0.3) |
| **Demographics** |  |  |  |  |  |  |  |  |  |  |
| None | 1475 | (71.6) | 380 | (18.5) | 450 | (21.9) | 388 | (18.8) | 257 | (12.5) |
| Age only | 267 | (13.0) | 47 | (2.3) | 92 | (4.5) | 50 | (2.4) | 78 | (3.8) |
| Sex only | 87 | (4.2) | 36 | (1.7) | 20 | (1.0) | 20 | (1.0) | 11 | (0.5) |
| Gender only | 0 | (0) | 0 | (0) | 0 | (0) | 0 | (0) | 0 | (0) |
| Ethnicity only | 8 | (0.4) | 1 | (0.0) | 3 | (0.1) | 0 | (0) | 4 | (0.2) |
| Age + sex | 211 | (10.2) | 16 | (0.8) | 30 | (1.5) | 36 | (1.7) | 129 | (6.3) |
| Age + ethnicity | 2 | (0.1) | 0 | (0) | 0 | (0) | 0 | (0) | 2 | (0.1) |
| Sex + ethnicity | 8 | (0.4) | 0 | (0) | 0 | (0) | 0 | (0) | 8 | (0.4) |
| Age + sex + ethnicity | 1 | (0) | 0 | (0) | 0 | (0) | 0 | (0) | 1 | (0) |
| **Health outcomes** |  |  |  |  |  |  |  |  |  |  |
| None (N) | 1362 | (66.1) | 288 | (14.0) | 338 | (16.4) | 355 | (17.2) | 381 | (18.5) |
| Yes (Y) | 697 | (33.9) | 192 | (9.3) | 257 | (12.5) | 139 | (6.8) | 109 | (5.3) |

## Appendix VI: Distribution of recommendations by their types and outcome measures

|  |  | | **Type of recommendations** | | | | | | | |
| --- | --- | --- | --- | --- | --- | --- | --- | --- | --- | --- |
|  | **Total** | | **Screening** | | **Diagnosis** | | **Management, Pharmacological** | | **Management, non-pharmacological** | |
|  | N | (%) | N | (%) | N | (%) | N | (%) | N | (%) |
| **Total** | 2059 | (100) | 174 | (8.5) | 224 | (10.9) | 802 | (39.0) | 859 | (41.7) |
| **Diseases included** |  |  |  |  |  |  |  |  |  |  |
| 1 | 1523 | (74.0) | 74 | (3.6) | 174 | (8.5) | 583 | (28.3) | 652 | (31.7) |
| 2 | 514 | (25.0) | 82 | (4.0) | 40 | (1.9) | 183 | (8.9) | 194 | (9.4) |
| 3 | 56 | (2.7) | 10 | (0.5) | 5 | (0.2) | 28 | (1.4) | 11 | (0.5) |
| 4+ | 24 | (1.2) | 8 | (0.4) | 5 | (0.2) | 8 | (0.4) | 2 | (0.1) |
| **Demographics** |  |  |  |  |  |  |  |  |  |  |
| None | 1514 | (73.5) | 84 | (4.1) | 162 | (7.9) | 558 | (27.1) | 671 | (32.6) |
| Age only | 277 | (13.4) | 17 | (0.8) | 28 | (1.4) | 156 | (7.6) | 66 | (3.2) |
| Sex only | 90 | (4.4) | 12 | (0.6) | 18 | (0.9) | 27 | (1.3) | 30 | (1.5) |
| Gender only | 0 | (0) | 0 | (0) | 0 | (0) | 0 | (0) | 0 | (0) |
| Ethnicity only | 8 | (0.4) | 4 | (0.2) | 0 | (0) | 3 | (0.1) | 1 | (0) |
| Age + sex | 217 | (10.5) | 52 | (2.5) | 16 | (0.8) | 58 | (2.8) | 85 | (4.1) |
| Age + ethnicity | 2 | (0.1) | 1 | (0) | 0 | (0) | 0 | (0) | 1 | (0) |
| Sex + ethnicity | 8 | (0.4) | 3 | (0.1) | 0 | (0) | 0 | (0) | 5 | (0.2) |
| Age + sex + ethnicity | 1 | (0.1) | 1 | (0) | 0 | (0) | 0 | (0) | 0 | (0) |
| **Health outcomes** |  |  |  |  |  |  |  |  |  |  |
| None (N) | 1362 | (66.1) | 159 | (7.7) | 202 | (9.8) | 440 | (21.4) | 561 | (27.2) |
| Yes (Y) | 697 | (33.9) | 15 | (0.7) | 22 | (1.1) | 362 | (17.6) | 298 | (14.5) |
